# Zebrafish Model in Ophthalmology to Study Disease Mechanism and Drug Discovery

**DOI:** 10.3390/ph14080716

**Published:** 2021-07-25

**Authors:** Yiwen Hong, Yan Luo

**Affiliations:** State Key Laboratory of Ophthalmology, Zhongshan Ophthalmic Center, Sun Yat-Sen University, Guangzhou 510060, China; hongyw@mail2.sysu.edu.cn

**Keywords:** zebrafish, eye, disease model, mechanism, drug candidate

## Abstract

Visual impairment and blindness are common and seriously affect people’s work and quality of life in the world. Therefore, the effective therapies for eye diseases are of high priority. Zebrafish (*Danio rerio*) is an alternative vertebrate model as a useful tool for the mechanism elucidation and drug discovery of various eye disorders, such as cataracts, glaucoma, diabetic retinopathy, age-related macular degeneration, photoreceptor degeneration, etc. The genetic and embryonic accessibility of zebrafish in combination with a behavioral assessment of visual function has made it a very popular model in ophthalmology. Zebrafish has also been widely used in ocular drug discovery, such as the screening of new anti-angiogenic compounds or neuroprotective drugs, and the oculotoxicity test. In this review, we summarized the applications of zebrafish as the models of eye disorders to study disease mechanism and investigate novel drug treatments.

## 1. Introduction

Until 2020, an estimated 295 million people suffer from the moderate and severe visual impairment worldwide, and among them, about 43.3 million people are even blind [1]. The leading global causes of blindness are cataract, followed by glaucoma, age-related macular degeneration (AMD), and diabetic retinopathy (DR) [2]. Visual impairment is a major concern of public health worldwide. The understanding of the pathological mechanisms involved in eye diseases is quite vital for the development of new therapeutics. Similarly, the available animal models characterized by closely mimicking eye pathology and allowing medium-throughput drug screening are desirable. Hence, model organisms with similar physiology to humans are essential to understand the developmental processes, identify potentially causative genes and factors for human eye disorders and develop the novel drug treatments [3].

Zebrafish *(Danio rerio)* is a kind of common aquarium fish originating from India and has become a prominent vertebrate model for studying diseases [4,5]. Zebrafish is more phylogenetically distant from humans than rodent, but it has 82% orthologues of human disease-associated genes [5]. In addition, zebrafish has a short generation time of 2–4 months, is productive with a single mating pair producing around 200 offspring at weekly intervals, and is easy to maintain at a low cost [6]. Importantly, the transparent zebrafish embryo develops ex utero, making the visualization of early organogenesis possible. The zebrafish eyes are relatively large compared to its overall small-size body, which enables eye bud manipulation during the early embryogenesis. Therefore, the easy accessibility of genes and embryos in zebrafish, as well as the similarity of eye with humans, has made zebrafish a predominant model of different eye diseases to elucidate their mechanism and investigate new treatments. 

In this review, we highlighted the use of zebrafish in modeling eye diseases by (i) introducing the characteristic anatomy and development of zebrafish eye, (ii) summarizing zebrafish models of eye diseases, such as cornea dystrophy, cataract, glaucoma, ocular vascular diseases, and photoreceptor degeneration, and (iii) presenting contributions of these models in the investigation of new drug candidates for eye diseases.

## 2. Anatomy and Development of Zebrafish Eye

Although zebrafish eyes are very small compared with those of humans, they contain almost all the basic structures of human eyes. Firstly, we focused on the anatomy and development of the zebrafish eye to explain why zebrafish is a promising model for human eye diseases.

### 2.1. Anatomy of Zebrafish Eye

#### 2.1.1. Cornea

Both of the zebrafish and human cornea contain five major layers: the epithelium, Bowman’s layer, stroma, Descemet’s membrane, and endothelium. In its mature state, the zebrafish corneal epithelium is 12.5 μm thick with four to six cell layers. The stroma is approximately 6 μm thick with 34 to 40 layers [7]. The endothelium, Bowman’s layer, and Descemet’s membrane are well developed. Several polypeptides highly enriched in the epithelium or the stroma of zebrafish cornea are the excellent markers of corneal differentiation [7]. Despite the similarities, the zebrafish ocular surface is dramatically different from the human, such as the absence of corneal nerve fibers, the thinner stroma, and the presence of rodlet cells [8].

#### 2.1.2. Iridocorneal Angle

Iridocorneal angle, the region where the cornea meets the iris, hosts cells specialized in maintaining intraocular pressure (IOP) [9]. IOP is balanced by the production and drainage of aqueous humor. Although the zebrafish ciliary epithelium lacks folds and processes, it still produces aqueous humor [9]. The servo-null electrophysiology is used to measure IOP in the anesthetized adult zebrafish as follows: when a pulled-glass microelectrode penetrates the cornea into the anterior chamber, the pressure transduction can be recorded [10]. Although the trabecular meshwork and aqueous humor dynamics of zebrafish are quite different from those of humans, the overall similarities in the average IOP and outflow tissue structure of aqueous humor make zebrafish a great model to investigate the complicated genetics of human glaucoma.

#### 2.1.3. Lens

Almost all the morphological features of human lens can be observed in the zebrafish eye [9]. The fish lens is more spherical than the human lens, and it is made up of an outer epithelial layer covering the elongated fiber cells [11]. Most lens epithelial cells are quiescent, except for a band of cells encircling the marginal equator, which finally proliferate and differentiate into fiber cells. Lens fiber cell differentiation happens in the transition zone, where elongating fiber cells lose their internal organelles and aid in the transparency [12]. In both the human and zebrafish, three kinds of lens crystallins are found: α, β, and γ. The similarities with human lenses make zebrafish an excellent model for studying crystallins in a living animal using the live embryo imaging.

#### 2.1.4. Visual System

**Figure 1:**
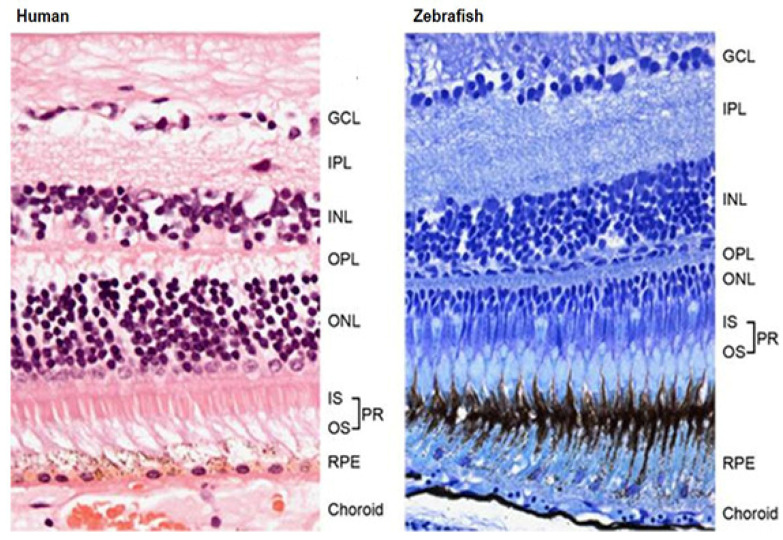
Cross-sectional view of the human and zebrafish retina indicating the similar structural features of the retinal layers [6].

Zebrafish is visually responsive at 72 h post fertilization (hpf), when the retina mirrors adult human retinal morphology and function [7]. Zebrafish retina is made up of an outer nuclear layer (ONL), outer plexiform layer (OPL), inner nuclear layer (INL), inner plexiform layer (IPL), and ganglion cell layer (GCL). It also possesses the same broad classes of retinal neurons in humans, such as retinal ganglion cells (RGC), bipolar cells, horizontal cells, and amacrine cells, and the same glial elements including Müller cells, astrocytes, and microglia (Figure 1) [6,13]. Furthermore, the zebrafish retina is cone-rich and analogous to the human macula, which results in good color vision and high-acuity vision [13]. In addition, zebrafish has four types of cones: blue (*sws2*), ultraviolet (*sws1*), green (*rh2*), and red (*lws*), among which the green and red cones exist as a physically fused double cones [13,14]. Visual signals arising from the photoreceptors are transmitted through the whole retina to the ganglion cells and subsequently transferred to the brain [3].

Zebrafish visual acuity is typically measured using the behavioral tests, including optokinetic response and optomotor response. The optokinetic response is a robust behavior in which moving objects evoke the tracking eye movements [15]. It is one of the most widely studied behaviors, due to its reliability and performance even in the immobilized larvae. Additionally, the optomotor response is a robust visual response in larval zebrafish, which is mediated by the red/green cone pathway [16]. When presented with a whole field moving stimulus, fish will turn and swim in the direction of perceived motion. Larvae can perform this behavior when swimming freely or when restrained by embedding their head in agar.

#### 2.1.5. Vasculature System

**Figure 2:**
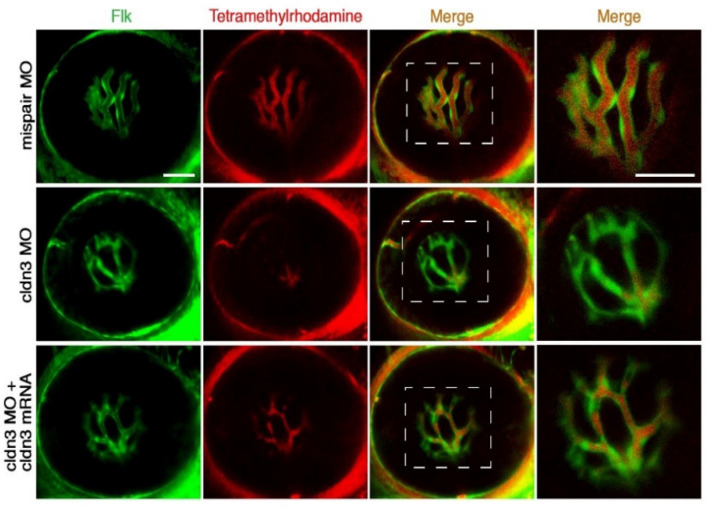
Lumen formation analyses of the hyaloid vessels in the 5 dpf zebrafish. More severe filling defects (more non-perfusion areas) of hyaloid vessels were observed in the cldnh MOs-injected zebrafish as compared to the mispair MO-injected or the cldnh mRNA-rescued zebrafish, indicating lumenization defects of the hyaloid vessels in the cldnh knockdown group. Scale bar: 50 μm [22].

The basic vascular biology of the developing zebrafish embryo is analogous to that of other vertebrates, and angiogenesis also plays a vital role in the zebrafish hyaloid vessel, which is similar to the development of retinal vasculature in mammalian embryos [17,18]. The primary zebrafish retinal vasculature branches from the central retinal artery by angiogenesis between 24 and 29 hpf. The optic artery goes into eye in a ventral direction through the optic fissure and forms a hyaloid loop, which exits the choroid fissure as the hyaloid vessel [6]. After passing through the choroid fissure, this artery system forms a network on the lens before 5 days post fertilization (dpf) [17,19]. The hyaloid vessels branch and adhere to the inner limiting membrane of the juvenile retina by 30 dpf, unlike the regression observed in humans [19,20,21]. Knockdown of some genes encoding crucial proteins involved in angiogenesis, such as cldnh, can interrupt the lumenization of the hyaloid vessels (Figure 2) [22]. Recently, new imaging techniques such as in situ hybridization for vascular-specific genes, dye injection-based vessel visualization, and the functional manipulation of vasculature in zebrafish embryos make zebrafish an exciting model for investigating human ocular vascular diseases [18]. In addition, more and more transgenic lines expressing fluorescent reporter proteins in the vascular system have emerged to help to study vascular diseases, such as the Tg *(fli1: EGFP)* line, which expresses EGFP under the control of *fli1* regulatory sequences [22,23,24].

### 2.2. Development of Zebrafish Eye

**Figure 3:**
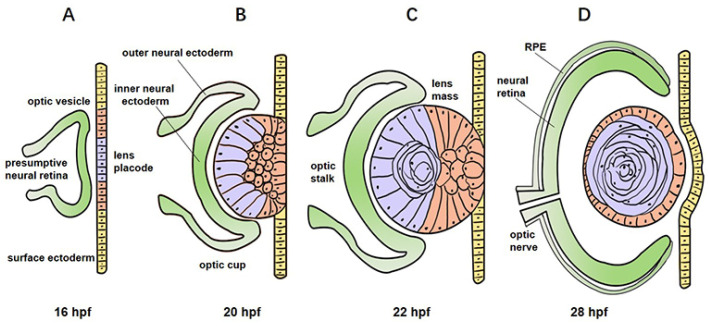
Schematic diagram indicating the development of zebrafish eye. The optic vesicle and lens placode are formed as the central eye field splits at 16 hpf (**A**). The distal portion of the optic vesicle invaginates so that a double-walled cup structure composed of an inner and outer neural ectoderm can be seen by 20 hpf (**B**). The optic cup grows circumferentially. The lens placode gives rise to a solid lens mass due to progressive delamination of the surface ectoderm cells by 22 hpf (**C**). The inner layer of the optic cup gives rise to the neural retina, while the outer layer differentiates into the RPE by 28 hpf (**D**). Finally, cells of the central lens placode move to the posterior lens mass and give rise to primary lens fiber cells (purple); cells of the peripheral lens placode migrate to the anterior lens mass to form the anterior epithelium (orange); the cornea (yellow) develops when the lens placode closes after the lens mass, taking apart from the surface ectoderm.

The development of zebrafish eye is strongly similar with that of humans and other vertebrates [25,26]. They all develop from three distinct embryological layers: surface ectoderm forming the lens and subsequently the corneal epithelia; neuroectoderm forming the neural retina, retinal pigment epithelium (RPE), optic stalk, and ciliary body; and neural crest cell-originated mesenchyme forming the corneal endothelium and stroma, iris stroma, vasculature, and sclera. Here, we drafted a schematic diagram indicating the development of zebrafish eye according to some studies in the literature (Figure 3) [6,9].

The development of zebrafish eye is fairly rapid. Neurogenesis begins at 28 hpf, and zebrafish embryos possess visual function as early as 72 hpf [27,28]. The optic vesicle, which finally gives rise to the neural retina and the RPE, evaginates from the forebrain at around 12 hpf and remains attached to the forebrain through a transient structure called the optic stalk [3]. After a series of morphogenetic events, the optic vesicle gives rise to the optic cup at 16 to 20 hpf, forms ventrally the optic fissure by 24 hpf, and later closes by 48 hpf [29]. The lens placode delaminates from the surface ectoderm cells overlying the optic cups at 16 hpf [6,30], forms a solid lens mass at approximately 24 hpf, and fully detaches from the surface ectoderm by the apoptosis of the intervening cells by 28 hpf [11,31,32]. At 30 hpf, the surface ectoderm that does not form the lens begins to possess corneal epithelial identity, and it forms migratory periocular mesenchymal cells migrating into the cornea from the peripheral regions of the optic cup between 30 and 36 hpf [7,33].

## 3. Zebrafish as a Model for Studying Mechanisms of Eye Disorders

With genetic accessibility, similar characteristics of human ocular development and easy controllability of living environment zebrafish has been used as a popular model to study eye disorders. Over the past decades, many human ocular diseases, such as cataract, glaucoma, DR, and AMD, have already been modeled in zebrafish. Here, we briefly introduced some important zebrafish models of eye diseases from anterior segments to posterior segments.

### 3.1. Corneal Dystrophy

Corneal dystrophies have a variable age of onset, variable inheritance, and progressive effects on corneal transparency and vision [34]. Due to the thinner stroma and presence of rodlet cells in the zebrafish cornea, zebrafish is seldom utilized to study the normal or pathological human corneas. Meanwhile, zebrafish has been used to study the function of genes whose mutation cause corneal dystrophy due to its genetic accessibility. Some genes responsible for human corneal dystrophy, such as *pip5k3, col17a1,* and *keratocan*, also express in the zebrafish cornea. *pip5k3* and *col17a1* are quite conservative without ocular alteration, while *keratocane* is significant for corneal transparency and structure [35,36,37]. Likewise, the loss of *lama1*, a gene encoding an important basal membrane protein, leads to focal corneal dysplasia in zebrafish [38]. Overall, probably due to some structural difference from humans, only four of the genes mentioned above have been reported to study corneal dystrophy in zebrafish models, which is quite few compared with mouse models [8]. Therefore, the use of zebrafish to study human corneal diseases should be undertaken with some particular caution.

### 3.2. Cataract

Cataract, which is characterized by cloudy vision due to lens opacity, mainly includes congenital cataract and age-related cataract (ARC). Genetic studies have identified over 30 causative mutation genes for congenital or other early-onset forms of cataract and only few ARC-associated gene variants [39]. Nevertheless, the causative genes for many cases of cataract remain unidentified. When causative genes of human cataract are knocked down in zebrafish embryos, cataract or other lens abnormalities are often present (Table 1). Therefore, zebrafish is a promising animal model to reveal the specific mechanism involved in cataract formation.

The use of zebrafish cataract model mainly focuses on the congenital cataract. Among the known gene mutations caused cataracts, mutations in lens crystallins account for the majority, followed by mutations in various growth or transcription factors, connexins, membrane proteins, and lipid metabolism [40]. Hence, the mechanism of crystalline in cataract formation has been deeply investigated. Recently, scientists have identified the function of a cataract-causing gene using the zebrafish model. Mutation of the *CRYAB* gene, a member of α-crystalline, results in congenital cataract by activating glucocorticoid receptor signaling [41]. Similarly, the *cloche* mutant in zebrafish displays cataract related to the insolubility of *γ-crystalline* and the faulty differentiation of lens fiber cells [42]. Meanwhile, the cataract phenotype can be rescued by the overexpression of *αA-crystalline* in the *cloche* mutant. Therefore, the *cloche* mutation can be used to investigate the aggregation of lens crystallin and prevent cataract. In addition, the overexpression of *Pax6*, which is a paired box and homeobox domain protein expressing in the developing nervous system and eye, causes defects in lens fiber cells and human congenital cataract. The *Pax6* mutant in zebrafish shows the alterations in the eye size and the abnormalities in lens differentiation [39,43,44]. The mutation of *Pitx3* and *Foxe3* genes can cause the mesenchymal dysgenesis of anterior segment and cataracts in humans, and the knockdown of *Pitx3* and *Foxe3* in zebrafish via antisense morpholino results in the lens dysmorphogenesis [45,46,47,48,49]. Interestingly, the mutations in *Hsf4*, a member of the heat-shock transcription factor family, lead to isolated cataracts in humans and an early onset cataract with multiple developmental defects in zebrafish lens by interrupting the terminal differentiation of a lens fiber cell [40,50]. 

ARC also has a genetic component, which makes individuals with the variation more vulnerable to environmental insults and aging [51]. Since the very old, even 2.5-year-old, zebrafish lens is not cloudy at all, zebrafish is not a predominant organism for modeling ARC [52]. However, zebrafish is a wonderful tool to investigate the mechanisms of crystalline-involved ARC. The chaperone capacity of *α-crystallins* is considered to be titrated out by the binding of the damaged lens proteins as well as the truncation and insolubilization of the small heat shock proteins; so zebrafish is used to establish a link between in vitro mechanistic models of *α-crystallin* chaperone and their roles in lens aging [53,54]. Additionally, mutation in the *CRYGC* gene *(γC-crystalline)* causes a remarkable reduction in the thermal stability of *γC-crystalline* and raises the risk of lens opacity when exposed to heat and UV-irradiation stresses, finally resulting in cataract [55]. 

### 3.3. Glaucoma

Glaucoma is a kind of optic neuropathy characterized by the progressive and irreversible visual field loss and visual impairment secondary to RGC loss [66]. Primary open angle glaucoma (POAG) without a certain physical cause explains 75–90% of glaucoma and possesses RGC loss, which is the common hallmark of other glaucoma phenotypes [67]. Other forms of glaucoma have also been classified, including primary closed angle glaucoma (POCG), developmental glaucoma, pigmentary glaucoma, steroid-induced glaucoma, etc [10,68,69].

Zebrafish offer great chances to test specific hypotheses associated with glaucoma [9]. For instance, zebrafish have been utilized to demonstrate that *SIX6* variants interrupt the development of the neural retina and result in a decreased number of RGC and an increased risk of glaucoma-associated visual impairment [70,71,72]. As another instance, zebrafish has been used to study the function of *FOXC1*, which is one of the few well-established genes related to POAG [73]. The transcription factor *FOXC1* has been found as a vital mediator that reacts to oxidative pressure and suppresses apoptosis in cells associated with aqueous humor dynamics [74,75]. Furthermore, the *Bugeye* mutant, which develops high IOP, enlarged eye globes, morphological abnormalities, and functional deficits in the retina, is identified as the model for myopia and glaucoma [76,77]. Many zebrafish models for glaucoma have been recently established based on human glaucoma (Table 2). However, all the mutations mentioned above do not relate to POCG, which is possibly because POCG is a complex heterogeneous disease that cannot be modeled by a single gene mutation.

Neurodegeneration in the form of RGC death is well documented in glaucoma. The chemical or oxidative stress-induced retinal damage is related to RGC injury and used in zebrafish research [28]. For example, NMDA, an analog of L-glutamate and an excitatory neurotransmitter in the mammalian central nervous system, can induce cellular excitotoxicity and RGC loss, and then glaucoma and retinal neurodegeneration [78]. However, the NMDA-induced neurotoxicity model only focuses on a sole mechanism of glutamate excitotoxicity in glaucoma pathology. Since the pathogenesis of glaucoma in humans is more complicated, this model may not thoroughly demonstrate the disease process, but the NMDA injection is a feasible choice for normal-tension glaucoma [79]. Additionally, oxidative injury plays an essential role in glaucoma onset as well as an imbalance between pro-oxidant and antioxidant capacities [80]. Therefore, the intravitreal injection of hydrogen peroxide in 5 dpf zebrafish larvae is used to establish the glaucoma model [81]. Nevertheless, a major flaw of utilizing zebrafish to study glaucoma is the notable capacity for retinal cell regeneration, including the GCL [81,82]. In summary, the zebrafish models of glaucoma chiefly shed light on POAG associated with gene mutation, and on RGC injury.

### 3.4. Vascular Disease

Pathological retinal angiogenesis makes a great contribution to irreversible causes of visual impairment at all ages, such as retinopathy of prematurity (ROP), DR, and AMD. Due to the significant similarities of vasculature between zebrafish and human, zebrafish embryos have been used for the identification of genes and mechanisms involved in pathological retinal angiogenesis.

#### 3.4.1. Diabetic Retinopathy

The crude prevalence of blindness caused by DR shows a global increase in age-standardized prevalence in 2020 [2]. DR is a common microvascular complication of diabetes, with manifestations of vision loss or blindness caused by the damage to retinal blood vessels. Animal models, such as mouse models and rat models, have been used to investigate the pathogenesis of DR and discover novel drugs. There is a rising interest in the DR zebrafish model due to their similar retinal vascular pathology and glucose metabolism as humans [93].

Retinal abnormalities of hyperglycemic zebrafish are consistent with those of diabetic patients. After being immersed alternately in glucose solution and water for 28 days, zebrafish has a remarkably thinner IPL and INL [94]. Moreover, hyperglycemia influences the cone photoreceptor neuron layer [95]. Further study shows that zebrafish retinal electrophysiology is adversely affected by the prolonged hyperglycemia, with separate actions in both distal and proximal retina [96]. Additionally, a recent study describes a novel, short-term, in vivo screening method for compounds affecting DR by exposing adult zebrafish to hyperglycemia conditions [97]. After treating with 130 mM glucose from 3 to 6 dpf, the zebrafish embryos show the dilation of hyaloid-retinal vessels as well as the increased levels of vascular endothelial growth factor (VEGF) at 6 dpf [97]. The *pdx1* mutants in zebrafish provide the only known model in which hyperglycemia-induced retinal angiogenesis can be studied [98]. Therefore, these zebrafish models have a realistic prospect in screening new drug candidates for DR treatment [99,100].

#### 3.4.2. Retinopathy of Prematurity

ROP is a kind of retinal vasoproliferative disease in premature infants and one of the leading causes of childhood blindness [101]. As ROP is a developmental disease, the zebrafish embryos can be a potential model for rapidly evaluating pharmaceutical treatments with a huge sample size in a short time span [102]. The zebrafish ROP model is established as follows: treating the Tg*(fli1:EGFP)* zebrafish with a hypoxia-inducing agent, followed by GS4012 (a VEFG inducer) at 24 hpf; then, the number of sprouts and vascular branches dramatically grow in the central retinal vascular trunks [102]. In addition, exposed to 10% air-saturated water for 3–12 days, adult Tg*(fli1:EGFP)* zebrafish can also develop severe retinal vascular proliferation [103].

#### 3.4.3. Age-Related Macular Degeneration

AMD, which blur the central vision, is a disorder with multifactorial pathogenesis, including angiogenesis, dysregulation in the complement, lipid, inflammatory, and extracellular matrix pathways [2,104]. Neovascular AMD, the subtype responsible for most of the vision loss, is characterized by the choroidal neovascularization in the macular area. The hypoxia-induced retinopathy model in mature zebrafish can be used to investigate neovascular AMD [103]. Similarly, the inactivation of the *von Hippel-Lindau (VHL)* gene promotes hypoxia-inducible factor signaling and consequent VEGF expression [18]. Accordingly, severe neovascularization of the choroid and hyaloid vessels, as well as retinal detachment and macular edema, have been noted in the *VHL* knockout zebrafish embryos [105]. Hence, *VHL* mutant zebrafish can be a model for neovascular AMD.

Dry AMD, the subtype characterized by RPE disorder, can result in the loss of photoreceptor cells. Interestingly, the zebrafish with the *gnn* mutant displays the AMD-related degeneration of red cones at around 5 dpf [106]. Additionally, the overexpression of *HTRA1*, a protein involved in the pathophysiology of AMD, can induce an accumulation of lipofuscin and melanolipofuscin between the photoreceptor and RPE layers in zebrafish [28]. The transgenic overexpression of human *HTRA1* in zebrafish displays certain morphologic changes of the RPE, photoreceptor cell death, and lipofuscin accumulation, which are the features of early AMD [107]. Recently, the *RP1L1* mutant zebrafish using CRISPR/Cas9 genome editing is the first zebrafish model of photoreceptor degeneration with subretinal drusen deposits, which is a hallmark of AMD [108].

### 3.5. Photoreceptor Degeneration

Photoreceptor degeneration diseases are exceedingly various, creating the challenges of preventing or reversing vision loss. Due to the similarities in retinal anatomy and function between zebrafish and humans, the zebrafish model has become a predominant model for studying the photoreceptor development and disease. Here, we focused on retinitis pigmentosa (RP) and Leber congenital amaurosis (LCA), which are two major kinds of retinal degeneration diseases [109].

#### 3.5.1. Retinitis Pigmentosa

RP is a disease characterized by decreased night vision and loss of peripheral vision due to the progressive photoreceptor cell death and dysfunction of the photoreceptors. The most common cause of human autosomal RP is the mutation in the rod-specific opsin gene, *rhodopsin* (*RHO*). Recently, various *RHO* mutant zebrafish models associated with dominant or recessive RP have been established with progressive rod degeneration [110,111,112]. Importantly, cone photoreceptors in zebrafish are unaffected by *RHO* mutants, which is consistent with the features of human RP caused by the *RHO* mutation [110,111]. 

X-linked RP, whose major cause is the mutation in *retinitis pigmentosa 2 (RP2),* is characterized by the early onset and rapidly progressive vision loss before 40 years old in humans [113]. Knockdown of *RP2* in zebrafish results in a small eye phenotype, gradual loss of the photoreceptors’ outer segments (OSs), and defective photoreceptor function, mimicking human X-linked RP [114,115]. Furthermore, zebrafish mutant phenotypes can be rescued by injecting human RP2 mRNA, revealing the vital role for *RP2* in the pathogenesis of X-linked RP [116]. Additionally, the great ability to simulate the various phenotypes of human RP in zebrafish models (Table 3) has been proved invaluable in identifying the causative genes for RP.

#### 3.5.2. Leber Congenital Amaurosis

LCA is a kind of inherited retinal dystrophy disease responsible for early-onset childhood blindness with immense genetic heterogeneity [130]. Presently, there are at least 15 LCA-associated genes, including *CEP290*, *RPE65*, *CRB1*, *KCNJ13*, *GUCY2D*, *AIPL1*, *CRX*, *IMPDH1*, *LCA5*, *LRAT*, *RPGRIP1*, *SPATA7*, *RD3*, *RDH12*, and *TULP1*. *CEP290* mutant zebrafish displays an intracellular transport delay and a decreased visual perception, which is analogous to human LCA patients [131]. Similarly, the knockout of *LCA5* in zebrafish using CRISPR/Cas9 technology causes the impaired OS protein trafficking and then cone–rod dystrophy, which mimics the phenotype of cone–rod dystrophy in humans [132].

Mutation genes involved in ciliogenesis initiation and the transport of cilium components can result in LCA or an LCA-like phenotype in mouse models [133]. Intraflagellar transport proteins play vital roles in the movement of cargo in the cilium, which can be facilitated by kinesin motors [13]. For example, *ift28, ift88*, and *ift172* mutants of zebrafish have the rapidly degenerated photoreceptors and without the developed photoreceptor OS [134,135,136]. The *kif3a* (kinesin family 3a) mutant in zebrafish causes photoreceptors to dramatically degenerate and fail to develop OSs, resulting in the extinguished ERG in zebrafish larvae [122,137,138].

## 4. Zebrafish as a Model for the Drug Discovery of Eye Disorders

Since the drug candidates can be added to the water culture medium rather than injected into the fish, zebrafish has become a promising model for the various successful phenotype-based drug discovery [5,139]. Here, we mainly discussed the use of zebrafish in the research of anti-angiogenic compounds, neuroprotective drugs, and oculotoxicity.

### 4.1. Anti-Angiogenic Compounds

The chemical testing in zebrafish can screen for new anti-angiogenic drugs for the eye diseases, which is analogous to the in vitro/ex vivo platforms. Therefore, zebrafish has been emerging as an exciting new model organism to discover anti-angiogenic drugs for ocular diseases. For instance, the screening of approximately 2000 compounds reveals that four small molecules affect retinal vessel morphology but do not produce obvious changes in the zebrafish trunk vessels and the retinal neuronal architecture [140]. Similarly, a bioactive chemical library of 465 drugs has been screened to identify small molecule inhibitors for the hyaloid vasculature angiogenesis in zebrafish larvae, and the researchers found 10 effective compounds, among which VDR agonists are the most effective ones [19].

In a small chemical screen using zebrafish, LY294002, the PI3K inhibitor, is identified as an effective and selective inhibitor of ocular angiogenesis without systemic side effects and diminishing visual function [141]. Additionally, zebrafish can serve as an early model for testing anti-VEGF drugs by investigating the effect on angiogenesis and its cytotoxicity. The inhibitor of FGFR and VEGFR, brivanib, inhibits zebrafish embryonic angiogenesis without impairing neurodevelopment [142]. Furthermore, both sunitinib and ZM323881, the anti-VEGF agents, can effectively block hypoxia-induced neovascularization in zebrafish [103]. In addition, the VEGFR2 inhibitors, such as sunitinib and 676475, block the retinal neovascularization in *vhl* zebrafish [105,143]. A recent study also concludes that the orthogonal drug pooling strategy is a cost-effective, time-saving, and unbiased approach to discover novel inhibitors for the ocular angiogenesis in zebrafish larvae [144].

### 4.2. Neuroprotective Drugs

Zebrafish models of the photoreceptor disease provide a platform for discovering novel neuroprotective drugs. Zebrafish can be utilized as phenotypes in screening neuroactive compounds for photoreceptor degeneration [145,146,147]. An ENZO SCREEN-WELL REDOX library on a zebrafish autosomal dominant RP model finds that carvedilol, a beta-blocker, can increase the rod number and improve visual function [148]. Schisandrin B, an active component isolated from the traditional Chinese medicine (Fructus Schisandrae), is observed to improve light sensation in the *pde6c* zebrafish model of retinal degeneration [149].

The overactivation of histone deacetylases (HDACs) has been detected in models of photoreceptor degeneration, and HDAC6 inhibition may prevent neurodegeneration [150]. Moreover, it is important to note that HDACs inhibitors can also prevent photoreceptors from light injury-caused death [151]. In the *atp6v0e* mutant zebrafish model, a cone photoreceptor degeneration disease, HDAC6 inhibitors successfully reduce the number of apoptotic cells and improve the photoreceptor OS area and visual function [152,153,154]. Furthermore, HDAC6 inhibitions and the regulation of peroxiredoxin activity may play a significant role in protecting retinal cells and particular photoreceptors, indicating they are sufficient to rescue retinal cell death and visual function [153].

### 4.3. Drug Oculotoxicity

It is conceivable that many drugs possess oculotoxicity. The prolonged or high-dose exposure to a certain drug may cause eye damage and vision loss. Given that the vertebrate eye is highly conserved, zebrafish can be a useful model for studying the ocular toxicity of drugs [155]. Zebrafish as an efficient animal model can predict the adverse ocular effects at the preclinical stage [156]. In this study, a group of 3-dpf-old zebrafish larvae are treated with drugs for 2 days, and then, the visual behavior is assessed by visual motor response and optokinetic response. Five of the six known oculotoxic drugs, including digoxin, gentamicin, ibuprofen, minoxidil, and quinine, also show some adverse effects on the visual responses of zebrafish. However, zebrafish retina has a different reactivity pattern from mammalian animals against some typical retinal toxicants in terms of histopathology, such as sodium iodate and N-methyl-N-nitrosourea [157]. Overall, when demonstrating the utility for detecting oculotoxic chemicals, the zebrafish assays have a sensitivity and specificity of 68–83% and 75–100%, respectively [155]. In addition, the chronic exposure to medroxyprogesterone acetate, an action of progesterone, can result in the overgrowth of the eyes and the defective visual functions in zebrafish [158]. These findings suggest that zebrafish models are powerful in resembling oculotoxic characteristics of drugs in humans and predicting oculotoxicity profiles of novel drugs.

## 5. Conclusions

Zebrafish provides a convenient animal model for mechanism investigation and drug discovery in ophthalmology due to their similar eye structure with human and accessibility to genetic manipulation. In the last few years, genome editing technologies, particularly based on Crispr/Cas9, have made it fairly easy to generate lines of zebrafish with mutations in targeted genes [159]. Hence, we are looking forward to the more popular zebrafish model to fully understand the genetic basis of eye diseases in the near future. Zebrafish has been widely used in drug discovery in ophthalmology, such as the screening of new anti-angiogenic compounds or neuroprotective drugs, and testing oculotoxicity. Rapid advances in high-throughput phenotyping point to the promising applications for zebrafish in drug discovery. Zebrafish has become an increasingly attractive model for understanding various human eye diseases and screening new drugs, whose highlights and drawbacks were summarized in Table 4.

Zebrafish models might be more predominant, powerful, and promising tools for investigating the mechanisms of various human eye diseases and discovering the novel drug therapy in the future.

## Figures and Tables

**Table 1:** Genetic mutations of zebrafish models for congenital cataract.

Function	Mutant Gene	Ocular Phenotype	Reference
encoding crystallins	*CRYAA(αA-crystallin) *	crystal-like opacity sporadically spreading across the lens, or frequent droplets covering a large fraction of the lens	[54]
	*CRYAB(αB-crystallin)*	same as *CRYAA*	[41]
	*CRYGC(γC-crystalline)*	same as *CRYAA*	[55]
	*CRYGD(γD-crystallin) *	same as *CRYAA*	[56]
encoding developmental factors	*DNase1l1l *	retaining nuclei in lens fiber cells	[57]
	*epha2 *	smaller eye, lens opacification and coloboma	[58]
	*mab21l2 *	microphthalmia, colobomas, small and disorganized lenses, cornea dysgenesis	[33]
	*plod3 *	distorted and dislocated lenses from an early stage dislocated, lack of normal lens capsule	[59]
	*rbm24 *	coarse and irregular lens, small-size retina and lens	[60]
encoding membrane proteins	*aqp0a *	nuclear opacity and widespread cortical fiber-to-fiber membrane stacking defects	[61]
	*gja8 *	various sizes of lens opacity	[62]
	*kpna4 *	rugged and cloudy center part of the lens	[63]
	*pgrmc1 *	minor or mild nuclear central mass with fiber cell disorganization, and moderate or severe nuclear density with pitting	[64]
encoding transcription factors	*celf1 *	lens defects and cataract	[65]
	*foxe3 *	smaller eye and small, deformed or absent lenses	[49]
	*hsf4 *	cataract with overproliferation of the lens epithelial cells and excessive accumulation of fiber cells	[50]
	*pitx3 *	severe lens degeneration, lack of anterior chambers and outer segment structures	[45]

**Table 2:** Zebrafish models of glaucoma.

Method	Injury Paradigm	Ocular Phenotype	Model	Reference
**Gene-Targeted**	*cpamd8*	Iridocorneal angle hypoplasia	POAG	[83]
	*cyp1b1*	Neural crest migration into the anterior segment	POAG	[84]
	*foxc1*	RGC loss	POAG	[85]
	*gpatch3*	Anterior chamber angle hypoplasia and a decreased number of iridophores	POAG	[86]
	*guca1c*	RGC apoptosis	POAG	[87]
	*ocrl*	Defective cilia formation in Kupffer vesicles	POAG	[88]
	*pitx2*	Abnormal development of the cornea, iris, and iridocorneal angle	POAG	[89]
	*pmel*	Profound pigmentation defects and enlarged anterior segments	Pigmentary glaucoma	[90]
	*six6*	Smaller eyes and reduced number of RGC	POAG	[70]
	*Tg (Bugeye)*	Decreased retinal cell densities and diminished outer retinal function	POAG	[91]
	*wdr36*	Thinner retinal layers and smaller eyes	POAG	[92]
**Chemical-** **Induced**	N-Methyl-D-aspartic acid (NMDA)	RGC loss	Glaucoma	[78]
**Oxidative Stress-Induced**	hydrogen peroxide	RGC injury	Glaucoma	[81]

**Table 3:** Zebrafish models of retinitis pigmentosa-like diseases.

Gene	Photoreceptor Features	Reference
*adipor1*	Decrease in rod photoreceptors	[117]
*cerkl*	Photoreceptor functional defects at 7 dpf. Rod OS defects at 3 months, cone OS defects at 7 months. Notable thinning of the photoreceptor layer and cell death by 12 months	[118]
*dact2*	Photoreceptor disc membrane disarrangement at 5 dpf	[119]
*eys*	Progressive photoreceptor loss; cone degeneration at 6 months, rod degeneration at 14 months	[120]
*her9*	Decrease in rod photoreceptors at 5 dpf. Few double cones with short OSs at 12 dpf	[121]
*kif3b*	Delayed OS development. Rapid rod degeneration by 5 dpf	[122]
*myo7aa*	Decreased photoreceptor function at 5dpf. Reduced rods at 8 dpf	[123]
*poc1*	Decrease length of photoreceptor OSs at 4 dpf	[124]
*prom1*	Decrease in cone photoreceptors at 7 dpf. Longer rod Oss. Delayed development of OSs	[125]
*prpf31*	Decreased in neuronal precursors and mature neurons at both 48 and 60 hpf	[126]
*rho*	Rod loss observed at 6 dpf. Degeneration continues into adulthood	[112]
*rp1l1*	Rod dysfunction at 6 months. Subretinal drusenoid deposits at 11 months. Photoreceptor loss at 12 months	[108]
*rp2*	Photoreceptor functional defects at 7 dpf. Short rod OSs at 2 months; cone OS defects at 4 months; significant rod OS loss and decreased cone OSs by 7 months	[115]
*rpgrip1*	No rod OSs at 5 dpf. Cone dysfunction at 7 dpf. Severe rod degeneration by 3 months, followed by cone degeneration. Degeneration of most photoreceptors by 23 months	[116]
*slc7a14*	Decreased photoreceptor function at 5 dpf. Reduced rod photoreceptors and peripheral RPE at 5 dpf	[127]
*SNRNP200*	Photoreceptors loss at 3 dpf	[128]
*ush2a*	Decreased photoreceptor function at 5–7 dpf and increased photoreceptor apoptosis at 8 dpf. Notable rod OS degeneration at 12 months, cone OS degeneration at 20 months	[129]

**Table 4:** Highlights and drawbacks of zebrafish models for common ocular diseases.

Disease Model	Highlights	Drawbacks
Corneal dystrophy	Able to identify related specific gene mutations	Not suitable for modeling other corneal diseases
Cataract	Feasible to study disease mechanisms, especially those involved in crystallins	Unavailable to model ARC
Glaucoma	Available to test specific hypotheses associated with glaucoma	Unsuccessful at establishing POCG models
	Zebrafish bugeye mutant with high IOP	Regenerative capability of retinal neurons, especially RGC cells
	Able to induce model of RGC loss	
Vascular disease	Available to identify related genes and mechanisms	Regenerative capability of retinal neurons
	Transgenic zebrafish lines expressing fluorescent reporter proteins in the vascular system	
	The *pdx1* mutant zebrafish presenting hyperglycemia-induced retinal angiogenesis	Without ideal model for neovascular AMD
	Transgenic overexpression of human *HTRA1* zebrafish eye with the features of early AMD	
	Feasible to help screen new anti-angiogenic drugs	
Photoreceptor Degeneration	Available to have large array of functional and behavioral tests	Regenerative capability of retinal neurons
	Able to identify new neuroprotective drugs using large-scale discovery	
	Feasible to identify related mutations by genetic screens	

## Data Availability

Data sharing not applicable.
